# Supplementary material for: Deletion of wfs1 Impairs Oligodendrocyte Precursor Cells Dorsal Distribution and Myelination Through the wfs1-hmgcs1 Axis in Zebrafish
Source: Biology (Basel). 2026 Aug 16;15(16):1402. doi: 10.3390/biology15161402 (PMC13510151; doi:10.3390/biology15161402)
Supplement: Supplementary file 1 [file biology-15-01402-s001.zip › File S1-Transcriptome sequencing data and differentially expressed genes (DEGs) in wfs1-- zebrafish at 5 dpf/1.GO/wfs1KOvsWT/UP/wfs1KOvsWT.up.GOcc_DAG.pdf]

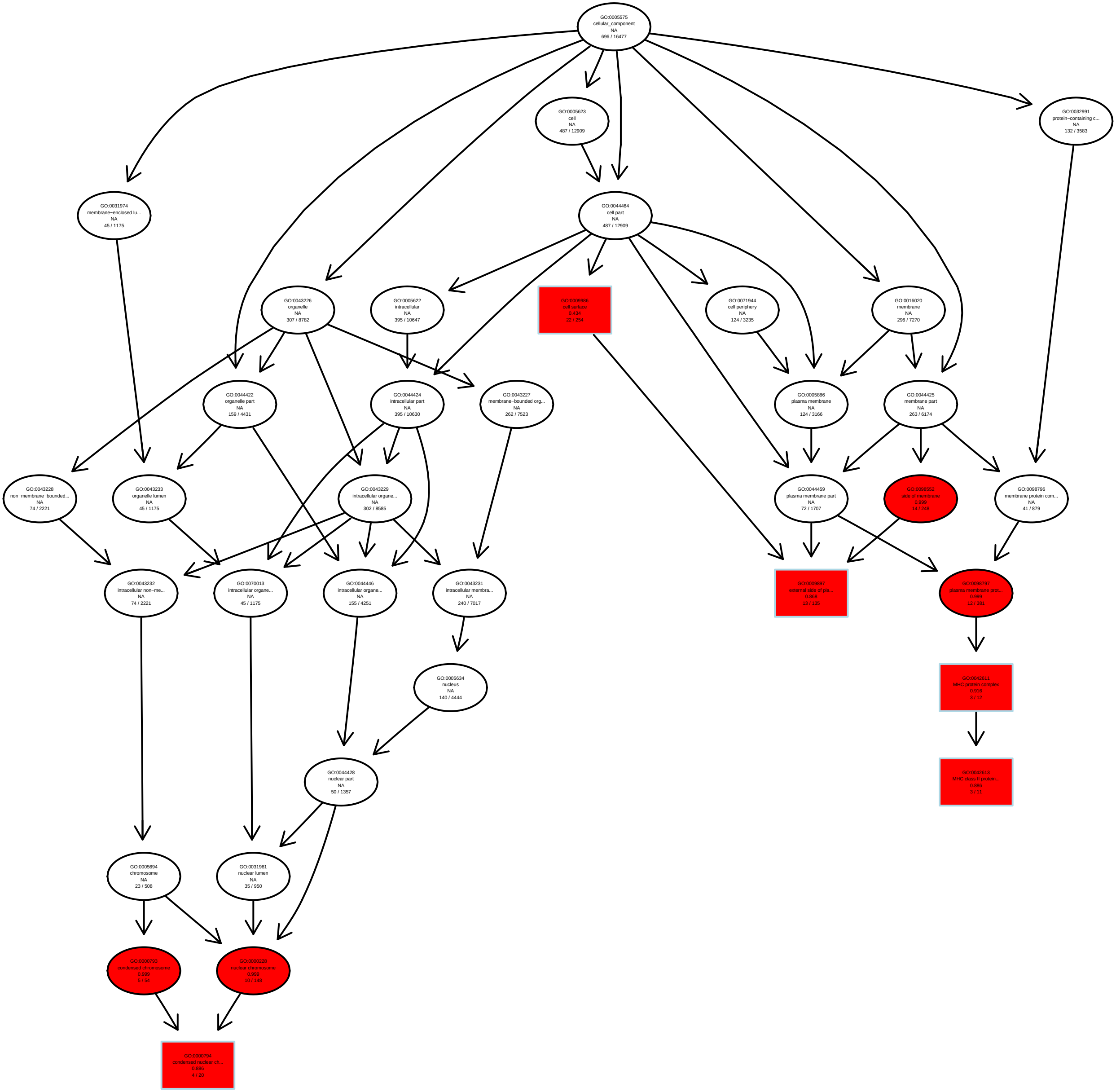
GO:0005575 cellular_component NA 696 / 16477
GO:0005623 cell NA 487 / 12909
GO:0032991 protein−containing c... NA 132 / 3583
GO:0031974 membrane−enclosed lu... NA 45 / 1175
GO:0044464 cell part NA 487 / 12909
GO:0043226 organelle NA 307 / 8782
GO:0005622 intracellular NA 395 / 10647
GO:0009986 cell surface 0.434 22 / 254
GO:0071944 cell periphery NA 124 / 3235
GO:0016020 membrane NA 296 / 7270
GO:0044422 organelle part NA 159 / 4431
GO:0044424 intracellular part NA 395 / 10630
GO:0043227 membrane−bounded org... NA 262 / 7523
GO:0005886 plasma membrane NA 124 / 3166
GO:0044425 membrane part NA 263 / 6174
GO:0043228 non−membrane−bounded... NA 74 / 2221
GO:0043233 organelle lumen NA 45 / 1175
GO:0043229 intracellular organe... NA 302 / 8585
GO:0044459 plasma membrane part NA 72 / 1707
GO:0098552 side of membrane 0.999 14 / 248
GO:0098796 membrane protein com... NA 41 / 879
GO:0043232 intracellular non−me... NA 74 / 2221
GO:0070013 intracellular organe... NA 45 / 1175
GO:0044446 intracellular organe... NA 155 / 4251
GO:0043231 intracellular membra... NA 240 / 7017
GO:0009897 external side of pla... 0.868 13 / 135
GO:0098797 plasma membrane prot... 0.999 12 / 381
GO:0005634 nucleus NA 140 / 4444
GO:0042611 MHC protein complex 0.916 3 / 12
GO:0044428 nuclear part NA 50 / 1357
GO:0042613 MHC class II protein... 0.886 3 / 11
GO:0005694 chromosome NA 23 / 508
GO:0031981 nuclear lumen NA 35 / 950
GO:0000793 condensed chromosome 0.999 5 / 54
GO:0000228 nuclear chromosome 0.999 10 / 148
GO:0000794 condensed nuclear ch... 0.886 4 / 20
